# Mendelian randomization study on the causal relationship between leukocyte telomere length and prostate cancer

**DOI:** 10.1371/journal.pone.0286219

**Published:** 2023-06-23

**Authors:** Bangbei Wan, Likui Lu, Cai Lv

**Affiliations:** 1 Reproductive Medical Center, Hainan Women and Children’s Medical Center, Haikou, China; 2 Department of Urology, Central South University Xiangya School of Medicine Affiliated Haikou Hospital, Haikou, China; 3 Institute for Fetology, The First Affiliated Hospital of Soochow University, Suzhou, China; Chengdu Medical College, CHINA

## Abstract

**Background:**

Leukocyte telomere length (LTL) is related to prostate cancer (PCa). However, the causal relationship between them remains unknown. This study was aimed at identifying the causal direction between LTL and PCa with Mendelian randomization (MR).

**Methods:**

Single-nucleotide polymorphisms associated with LTL were identified from a genome-wide association study (GWAS) involving 472,174 individuals. Summary-level data of PCa-related GWAS were extracted from four cohorts comprising 456,717 individuals. An inverse-variance-weighted (IVW) algorithm was used for MR. Sensitivity analyses were performed with MR-Egger regression, IVW regression, leave-one-out test, and MR-Pleiotropy Residual Sum and Outlier analyses. A meta-analysis was also performed to compute the average genetically determined effect of LTL on PCa.

**Results:**

A long LTL was associated with an increased risk of PCa in all cohorts, with odds ratios of 1.368 (95% confidence interval [CI]: 1.247 to 1.500, *P* = 2.84×10^−11^), 1.503 (95% CI: 1.243 to 1.816, *P* = 2.57×10^−5^), 1.722 (95% CI: 1.427 to 2.077, *P* = 1.48×10^−8^), and 1.358 (95% CI: 1.242 to 1.484, *P* = 1.73×10^−11^) in the IVW analysis. Sensitivity analyses showed that the genetically determined effect of LTL on PCa was stable and reliable. The meta-analysis showed that the genetically determined per 1-standard deviation rise in LTL correlated significantly with an average 40.6% increase in the PCa risk, with an average odds ratio of 1.406 (95% CI: 1.327 to 1.489, *P* < 0.001).

**Conclusion:**

The results of this study supported the causal hypothesis that the genetically determined longer LTL was associated with a higher risk of PCa. This finding could serve as a basis for therapeutic strategies for PCa.

## Introduction

Prostate cancer (PCa) is a common malignant neoplasm in old men, particularly in the Occident, and the second leading cause of cancer deaths among men [1]. Most cases of PCa are difficult to diagnose in the early stage because of the hidden onset [2]. The progression differs according to the pathological type and heterogeneity among cancer cells [3]. Currently, approximately 90% of patients show local tumor progression at the time of the PCa diagnosis, a contraindication for surgical treatments [4]. Therefore, diagnosing PCa at an early stage and implementing effective interventions improve the prognosis of patients with PCa.

Telomeres are complexes consisting of DNA of tandem TTAGGG repeats, ranging from several to 15 kilobases in length, and protein. They protect chromosomal and genetic stability [5, 6]. Changes in telomere length are strongly associated with aging and the occurrence and development of disease in humans [7–10]. During tumor progression, cancer cells can remain biologically active and proliferate by regulating telomere in the tumor [11, 12]. Changes in telomere length are closely related to the biological vitality of PCa cells and clinical characteristics of patients with PCa [13–15]. Thus, telomere length is correlated with the occurrence and progression of PCa. However, the causal relationship between them remains unclear.

Mendelian randomization (MR) is used to analyze the causal relationship between an exposure and its outcome using single-nucleotide polymorphisms (SNPs) as instrument variables [16, 17]. MR is similar to randomized controlled trials in overcoming the influence of residual confounding factors and superior in cost-effectiveness, ease of implementation, and time-consumption.

The aim of the present study was to utilise MR to elucidate the causal association between leukocyte telomere length (LTL) and PCa. The inverse-variance-weighted (IVW) algorithm was applied as the primary approach to illustrate potential causation. Reliability and robustness were tested using MR-Egger, weighted median, simple mode, and weighted mode methods. Considering the importance of LTL in human life, clarifying its potential causal impact on PCa could help prevent PCa. To the best of our knowledge, this is the first study to comprehensively investigate the LTL and the risk of PCa.

## Materials and methods

### MR study design

Summary-level data of genome-wide association studies (GWASs) were extracted from the IEU OpenGWAS database (https://gwas.mrcieu.ac.uk/), including four datasets related to PCa (outcome) and one dataset related to LTL (exposure). These datasets were utilized to actualize the two-sample MR analysis for illustrating the causal relationship between LTL and PCa.

### Assumptions for the MR study

**Fig 1:**
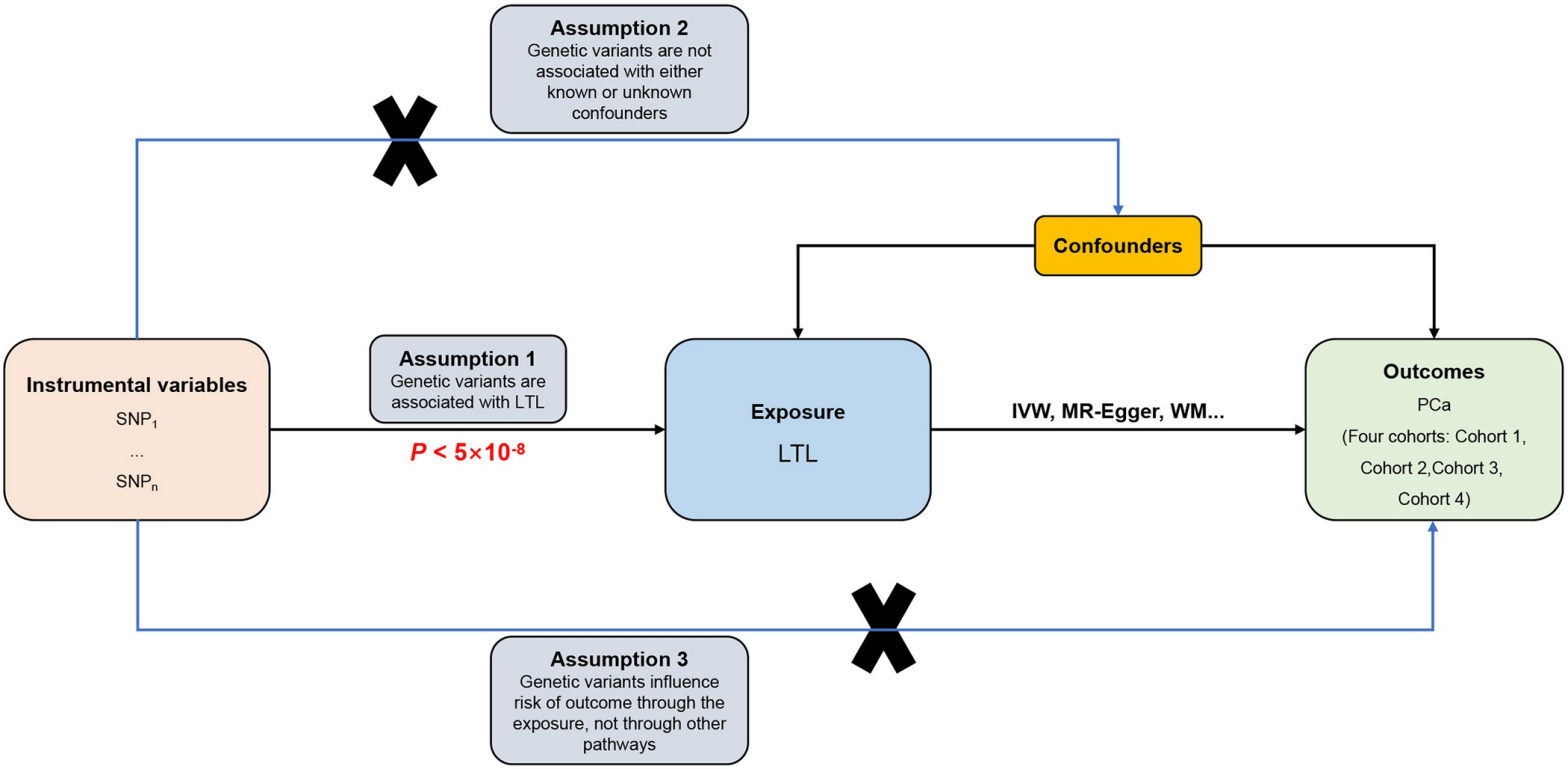
Flowchart of MR investigating the causal relationship between LTL and PCa. GIV assumptions: (1) GIVs must be strongly associated with LTL (*P* < 5×10^−8^); (2) GIVs must not be correlated with unmeasured confounders of the LTL and PCa relationship; (3) GIVs should only affect the risk of PCa through LTL. SNPs = single-nucleotide polymorphisms; LTL = leukocyte telomere length; PCa = prostate cancer; IVW = inverse-variance-weighted; WM = weighted median.

Performance of the MR study was set to conform with three fundamental assumptions. (1) Relevance assumption: Genetic instrument variables (GIVs) must be strongly associated with the exposure (s) of interest. (2) Independence assumption: All confounders of the correlations between GIVs and the outcome (s) should be measured. (3) Exclusion restriction: GIVs must affect the outcome only through their effect on the exposure (s) of interest. **Fig 1** shows the assumptions and design of the MR study.

### Data sources

Summary-level data of GWASs associated with LTL of the European population (472, 174 individuals from the IEU OpenGWAS Project cohort) were downloaded from the IEU OpenGWAS database (GWAS ID: ieu-b-4879) [18]. SNPs associated with LTL were extracted as GIVs based on these conditions: a genome-wide significant level (*P* < 5×10^−8^) and independence among SNPs (*r*^2^ < 0.001; clumping distance, 10,000 kb). To confirm the independence among SNPs, we used the PhenoScanner database (http://www.phenoscanner.medschl.cam.ac.uk/) [19] to judge and remove SNPs associated with confounders. To calculate the GIV power for MR, the F statistic was computed using the following formula: F = *R*^*2*^(N − K − 1)/K(1 − *R*^*2*^), where *R*^*2*^ denoted the proportion of the variance of the LTL explained by each GIV and its formula for calculation as follows: 2∗EAF∗(1 − EAF)∗β [20], where EAF represents the effect allele frequency of LTL, and β represents the estimated genetic effect of LTL; K denoted the number of included SNPs in MR; and N represented the sample size of LTL in GWAS. The GWAS summary statistics data associated with PCa from four European populations as outcomes were obtained from the IEU OpenGWAS database. To analyze expediency in MR, the datasets were classified as follows: cohort 1 (GWAS ID: ebi-a-GCST006085): 140,254 individuals (79,148 cases and 61,106 controls) from European Molecular Biology Laboratory’s European Bioinformatics Institute cohort [21]; cohort 2 (GWAS ID: finn-b-C3_PROSTATE): 95,213 individuals (6,311 cases and 88,902 controls) from the FinnGen biobank cohort; cohort 3 (GWAS ID: finn-b-C3_PROSTATE_EXALLC): 80,996 individuals (6,311 cases and 74,685 controls) from the FinnGen biobank cohort; and cohort 4 (GWAS ID: ieu-b-85): 140,254 individuals (79,148 cases and 61,106 controls) from the IEU OpenGWAS Project cohort.

### Bidirectional univariable MR analyses

To investigate the causality between LTL and PCa, a bidirectional univariable two-sample MR analysis was implemented for LTL and PCa as both exposure and outcome. Furthermore, the IVW [22] approach was used as the principal causal effect computing the pooled effect of all SNPs. MR-Egger [23], weighted median [24, 25], simple mode [25], and weighted mode [25] methods were used to validate the reliability and robustness of the results. The two-sample MR analysis may display heterogeneity because of discrepancies in analysis platforms, experimental conditions, inclusion populations, and SNPs, thereby impacting the estimation of causal effects. Therefore, the major IVW and MR-Egger algorithms were utilized to examine the heterogeneity. A *P*-value > 0.05 was regarded as no heterogeneity in the included GIVs, and the impact of heterogeneity on the estimation of causal effects could be negligible. Based on the aforementioned assumptions for the MR analysis, when a GIV directly influenced outcomes without impacting LTL, the fundamentals of MR were infringe. Finally, whether or not pleiotropy existed in the causal inference between LTL and PCa was tested. The Egger model’s intercept was adopted to estimate pleiotropy statistically; a deviation from 0 denoted the absence of directional pleiotropy [26]. The occurrence of pleiotropy in the analysis was identified using MR-pleiotropy residual sum outlier (PRESSO) [27, 28]. Pleiotropy in the MR analysis was unlikely at *P* > 0.05, and its effects were ignored. We employed the leave-one-out method and IVW and MR-Egger regression algorithms for sensitivity analyses. The directionality that LTL causes PCa was confirmed using the MR Steiger test, with statistical significance set as *P* < 0.05.

All MR analyses were implemented using the TwoSampleMR package version 0.5.6 in R version 4.1.2.

### Meta-analysis

To precisely calculate the average effect of genetically predicted LTL causing PCa, we performed a single-arm meta-analysis of the four PCa cohorts using the ‘meta’ package version 5.2.0. Heterogeneity among the casual effect from different cohorts was assessed using the chi-squared-based Q and *I*^2^ tests. A random- (*I*^2^ > 50%) or fixed-effects (*I*^2^ < 50%) model was utilized to pool the casual effect. Statistical significance was set as *P* < 0.05.

## Results

### Bidirectional univariable MR analysis results

To improve the reliability of the results, we selected four different PCa-related GWAS cohorts as the outcome to analyze the effect of LTL on PCa. A total of 134 independent SNPs associated with LTL were used as GIVs to conduct univariable MR. However, the number of GIVs ultimately included in the MR differs in the four cohorts. The reasons were as follows: (a) a portion of GIVs were missing in PCa-related GWAS summary-level data; (b) a portion of GIVs were removed because of pleiotropy. In cohort 1, a total of 134 independent SNPs associated with LTL were extracted from PCa-related GWAS summary-level data. Ten SNPs were then removed because of pleiotropy. The rest of 124 SNPs were included to investigate the effect size of the genetically predicted LTL on PCa. The IVW method showed an odds ratio (OR) of 1.368 (95% confidence interval [CI]: 1.247 to 1.500, *P* = 2.84×10^−11^). In cohort 2, all 130 independent SNPs associated with LTL were extracted from PCa-related GWAS summary-level data. 1 SNP was then removed because of pleiotropy. The remaining 129 independent SNPs were utilized to calculate the effect size of the genetically predicted LTL on PCa. The IVW method showed an OR of 1.503 (95% CI: 1.243 to 1.816, *P* = 2.57×10^−5^). In cohort 3, a total of 130 independent SNPs associated with LTL were extracted from PCa-related GWAS summary-level data. 2 SNPs were then removed because of pleiotropy. The rest of 128 independent SNPs were used to compute the effect size of the genetically predicted LTL on PCa. The IVW method showed an OR of 1.722 (95% CI: 1.427 to 2.077, *P* = 1.48×10^−8^). In cohort 4, all 134 SNPs associated with LTL were extracted from PCa-related GWAS summary-level data. 11 SNPs were then removed because of pleiotropy. The rest of 123 independent SNPs were applied to assess the effect size of the genetically predicted LTL on PCa. The IVW method showed an OR of 1.358 (95% CI: 1.242 to 1.484, *P* = 1.73×10^−11^). **Table 1** shows these results. The F statistics of all included SNPs were >10, indicating no weak-instrument bias (**S1 Fig** and **S1–S4 Tables**).

**Table 1:** MR results of LTL on the risk of PCa.

Outcome	Method	No. of SNPs	OR (95% CI)	*P*	*P*-het	*P*-intercept
PCa (cohort 1)	MR Egger	124	1.476 (1.240−1.757)	2.53×10^−5^	7.85×10^−7^	0.316
	Weighted median	124	1.414 (1.236−1.618)	4.56×10^−7^		
	**IVW**	**124**	**1.368 (1.247−1.500)**	**2.84×10** ^**−11**^	**7.09×10** ^**−7**^	
	Simple mode	124	1.391 (1.086−1.781)	0.009		
	Weighted mode	124	1.404 (1.222−1.614)	4.72×10^−6^		
	MR-PRESSO (raw)	124	1.355 (1.255–1.455)	2.68×10^−9^		
PCa (cohort 2)	MR Egger	129	1.451 (1.029−2.046)	0.036	0.004	0.81
	Weighted median	129	1.385 (1.064−1.803)	0.016		
	**IVW**	**129**	**1.503 (1.243−1.816)**	**2.57×10** ^**−5**^	**0.004**	
	Simple mode	129	1.171 (0.653−2.099)	0.597		
	Weighted mode	129	1.356 (0.972−1.893)	0.076		
	MR-PRESSO (raw)	129	1.500 (1.305–1.694)	4.32E-05		
PCa (cohort 3)	MR Egger	128	1.744 (1.243−2.447)	0.002	0.052	0.929
	Weighted median	128	1.540 (1.143−2.076)	0.005		
	**IVW**	**128**	**1.722 (1.427−2.077)**	**1.48×10** ^**−8**^	**0.059**	
	Simple mode	128	1.459 (0.805−2.642)	0.215		
	Weighted mode	128	1.553 (1.107−2.178)	0.012		
	MR-PRESSO (raw)	128	1.513 (1.908–0.969)	1.01×10^−7^		
PCa (cohort 4)	MR Egger	123	1.417 (1.198−1.677)	8.55×10^−5^	5.61×10^−5^	0.554
	Weighted median	123	1.411 (1.240−1.604)	1.63×10^−7^		
	**IVW**	**123**	**1.358 (1.242−1.484)**	**1.73×10** ^**−11**^	**6.42×10** ^**−5**^	
	Simple mode	123	1.417 (1.121−1.790)	0.004		
	Weighted mode	123	1.417 (1.247−1.610)	4.16×10^−7^		
	MR-PRESSO (raw)	123	1.344 (1.247–1.441)	2.18×10^−9^		

LTL = leukocyte telomere length; PCa = prostate cancer; IVW = inverse-variance-weighted; OR = odds ratio; *P*-het = *P*-value for heterogeneity using Cochran Q test; *P*-intercept, *P*-value for MR-Egger intercept; MR-PRESSO = Mendelian randomization-pleiotropy residual sum outlier; SNP = single-nucleotide polymorphism.

**Fig 2:**
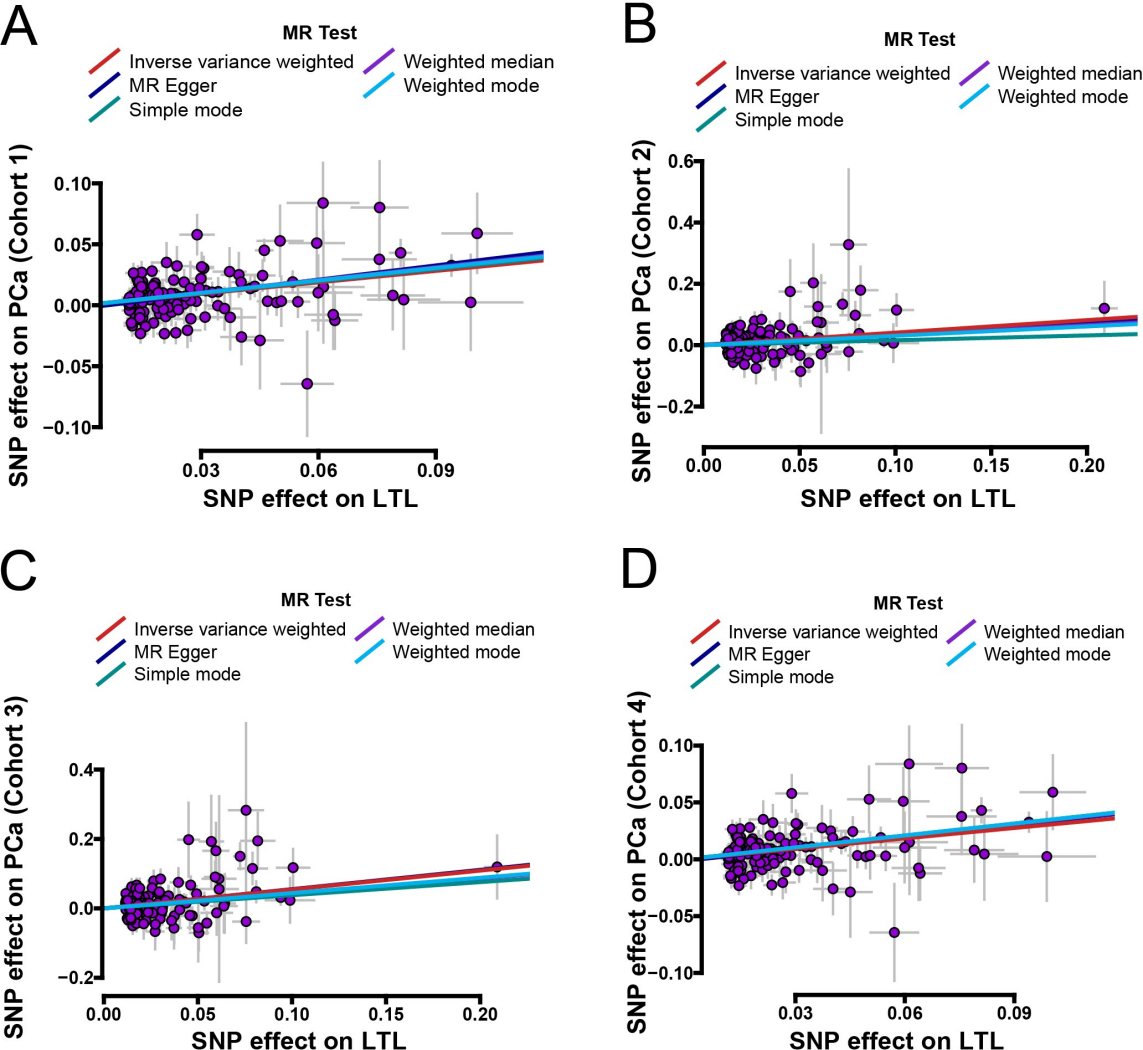
Scatter plots of LTL with the risk of PCa. **A–D,** Effect of LTL-related SNPs on the PCa risk from four different cohorts. Scatter plot demonstrating the effect of each LTL-associated SNP on PCa on the log-odds scale. Slopes of each line represent the causal association for each method. MR = Mendelian randomization; SNP = single-nucleotide polymorphism; LTL = leukocyte telomere length; PCa = prostate cancer.

**Fig 3:**
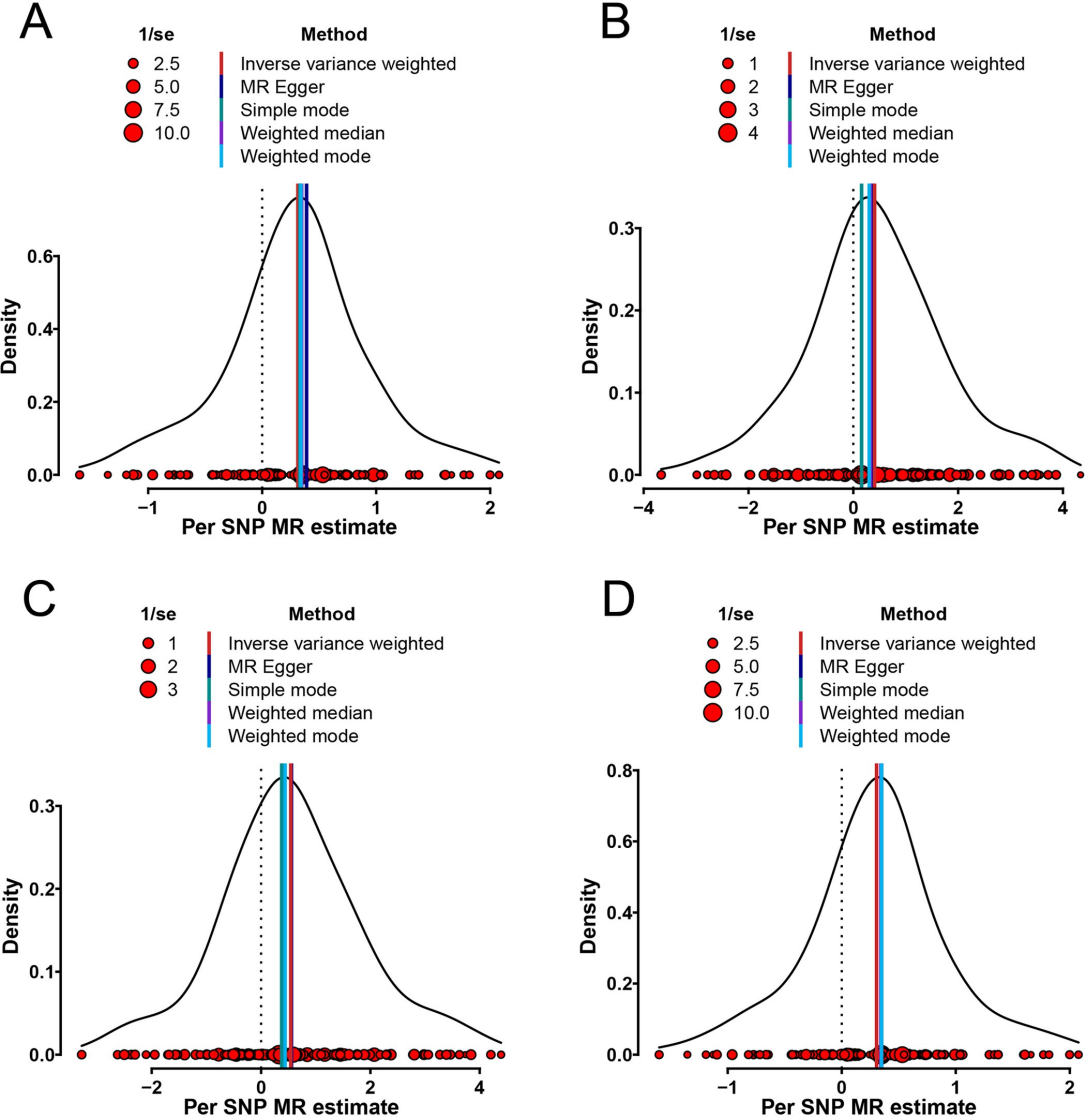
Density plots of LTL with the risk of PCa. **A–D,** Effect of LTL-related SNPs on the PCa risk from four different cohorts. **MR =** Mendelian randomization; **SNP =** single nucleotide polymorphism.

To test the reliability of these results, four algorithms, including MR-Egger, weighted median, simple mode, and weighted mode, were utilized to prove the causal direction from LTL to PCa. The results congruously supported that genetically predicted LTL increase was a risk factor of PCa (**Figs 2 and 3**).

Sensitivity analyses were performed to evaluate the stability of the aforementioned results. The Cochran’s Q test in the IVW (multiplicative random effects) (cohort 1: *P* = 7.09×10^−7^; cohort 2: *P* = 0.004; cohort 3: *P* = 0.058; and cohort 4: *P* = 6.42×10^−5^) and MR Egger (cohort 1: *P* = 7.85×10^−7^; cohort 2: *P* = 0.004; cohort 3: *P* = 0.052; and cohort 4: *P* = 5.61×10^−5^) models suggested heterogeneity in the instrumental variables of the three cohorts (cohort 1, cohort 2, and cohort 4), possibly resulting from true causality rather than violation of fundamental assumptions in the MR analysis. Statistical evidence from the MR-Egger intercepts and the MR-PRESSO global tests uniformly showed no horizontal pleiotropy. In addition, the leave-one-out analysis indicated that no SNP altered the combined estimate, supporting the stability and reliability of our results (**S2 Fig**). The causal assumption of LTL and PCa was proven by the MR Steiger test, and the impact of LTL on PCa was confirmed to be the correct causal direction (*P* < 0.001).

Moreover, reverse MR analyses were performed to verify the causal assumption of LTL and PCa. The results suggested no indication of reverse causality from LTL to PCa (**S3 Fig and S5 Table**).

### Meta-analysis results

**Fig 4:**
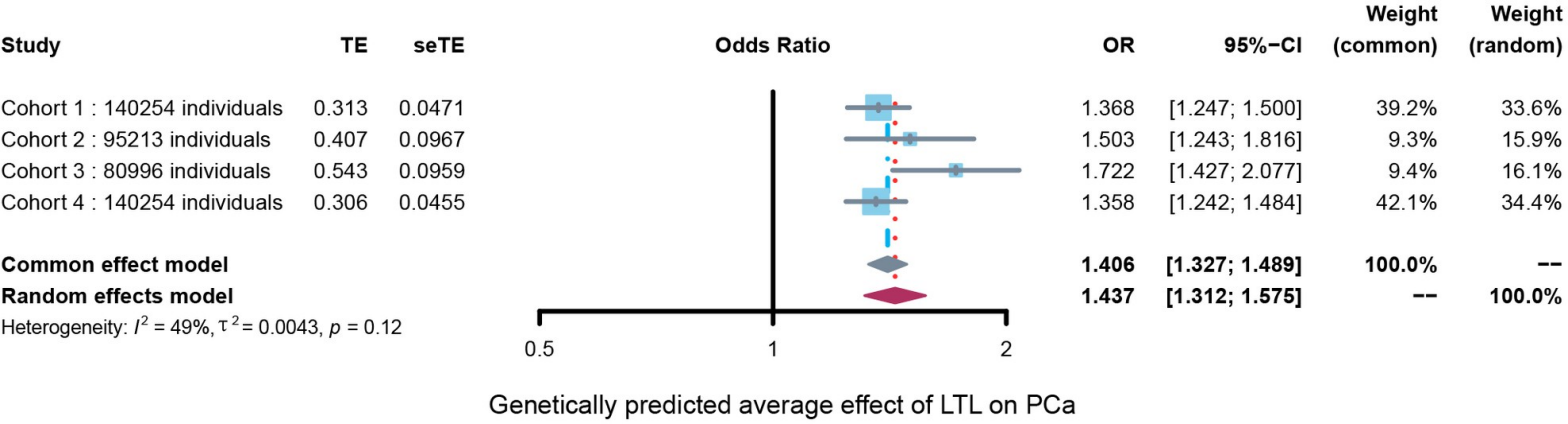
Forest plots to visualize the results of the meta-analysis of the four different cohorts. Forest plots demonstrating the average genetically determined effect of LTL on PCa. Presented OR and CI correspond to the average effects of LTL on PCa. *I*^*2*^ statistic and chi-squared-based Q were used to evaluate the heterogeneity among different studies.

The meta-analysis showed that a genetically predicted per 1-standard deviation rise in LTL correlated significantly with an average 40.6% increase in the PCa risk, with an average OR of 1.406 (95% CI: 1.327 to 1.489, *P* < 0.001). Heterogeneity among the four cohorts was evaluated using chi-squared-based Q and *I*^2^ tests. The results showed an acceptable heterogeneity among the four cohorts (Q = 5.86, *I*^2^ = 48.8%, *P* = 0.12). **Fig 4** shows the results of the meta-analysis.

## Discussion

In this study, using the most recent and largest GWAS data of the European ancestry, the combined MR and meta-analysis suggested that LTL impacting PCa was a distinct causality. The genetically predicted 1-standard deviation increase in LTL was significantly associated with an average 40.0% advance in the PCa risk.

Telomere is essential for maintaining chromosomal integrity, and its length is correlated with cancer risk and progression [28, 29]. In pancreatic cancer, an observational study involving 472 cases and 1,071 controls showed that LTL correlated negatively with the risk for pancreatic cancer [30]. Similarly, in colorectal carcinoma, LTL in normal adjacent tissues was longer than in cancer tissues, and longer LTL correlated positively with the good prognosis of patients and negatively with a higher tumor stage [31]. Furthermore, LTL plays a vital role in the occurrence and progression of PCa [31, 32]. A retrospective study involving 533 Austrian patients with PCa followed-up for a median duration of 149 months showed that LTL was closely related to the prognosis and that longer LTL predicted a lower overall survival rate [33]. In contrast, a recent study by Xu et al. [34] analyzed the association between LTL and aggressive PCa and showed shorter LTL in patients with PCa with higher Gleason scores and that shorter LTL correlated positively with biochemical recurrence of PC. The MR method confirmed that genetically predicted short LTL was related to an increased risk of biochemical recurrence of PCa. Similarly, a retrospective study involving 317 African American patients with PCa investigating the association between LTL and biochemical recurrence of PCa in patients who underwent radical prostatectomy and/or radiotherapy showed that shorter LTL correlated significantly with higher Gleason scores in patients with PCa. In addition, patients with PCa with shorter LTL were also distinctly associated with a higher risk of biochemical recurrence [35]. Despite these retrospective studies suggesting a correlation between LTL and the clinical characteristics and prognosis of patients with PCa, the causal relationship between LTL and the risk of PCa remains uncertain. In the present study involving over 400,000 Europeans showed that a longer LTL correlated with a higher risk for PC.

In a pan-cancer MR study, long LTL showed a significantly positive association with many cancers, including glioma, serous low-malignant-potential ovarian cancer, lung adenocarcinoma, neuroblastoma, bladder cancer, melanoma, testicular cancer, kidney cancer, and endometrial cancer. Although the results of this MR study suggested that a correlation between LTL and some cancers was not statistically significant, it preliminarily explained the trend of LTL influence on those cancers, such as basal cell carcinoma, breast cancer, colorectal cancer, PCa, esophageal cancer, pancreatic cancer, and head and cancer [36]. In addition, the study of Gao et al. [37] also reported that LTL was causally correlated with the risk of PCa. Although the above two MR studies also briefly illustrated the causal directional trend of LTL on PCa, the level of evidence was weak. The present study supported the causal relationship between LTL and PCa with a high level of evidence. First, the most recent and largest GWAS data of the European ancestry were utilized to perform statistical analyses. Second, LTL-related GWAS data from a large sample (472,174 individuals) were used to select effective instrumental variables, and all LTL data were standardized before analyses [18]. Third, the included SNPs were more comprehensive, thereby effectively avoiding the bias of an actual pooled effect in the analysis. Fourth, the statistical evidence from coherence and sensitivity analysis proved the stability and reliability of the results. Finally, the meta-analysis was used to further evaluate the genetically determined average effect size of LTL on PCa. Mechanistically, the effect of LTL on the risk of PCa partly attributes to overlong LTL seriously affecting immune cell function [34].

However, this study had some limitations. First, although our study suggested that a longer LTL correlated with a higher risk of PCa, a short LTL may increase the risks of other diseases. Therefore, we should carefully employ some interventions to prevent LTL in the short or long term. Second, the telomere length was detected in leucocytes, and whether or not it will reflect in the telomere length of other organ tissues is unclear. Third, GWAS data of the European population were utilized, with questionable generalizability to non-European ancestries. Finally, the potential biological mechanism of the effect of LTL on the risk of PCa is still unclear. Hence, more molecular experiment is necessary to validate the finding of this study.

In summary, this study provided powerful evidence to support the causal hypothesis that the genetically determined longer LTL was associated with a higher risk of PCa. In addition, because of genetic variants, the effect of LTL on PCa is lifelong. Therefore, our findings could serve as a basis for therapeutic strategies for PCa.

## Supporting information

S1 Fig Bar plots to visualize the F statistic for each GIV. **A–D,** Results of F statistics of the four cohorts.
(PDF)

S2 Fig Leave-one-out plots of LTL with the risk of PCa. Leave-one-out analysis for IVW MR of LTL on PCa in summary-level analyses. SNP = single-nucleotide polymorphism; LTL = leukocyte telomere length; PCa = prostate cancer; MR = Mendelian randomization.
(PDF)

S3 Fig Forest plot to visualize causal effects of variation in PCa on LTL. Presented OR and CI correspond to the effects of PCa on LTL (four cohorts). Results of MR using various analysis methods (MR-Egger, weighted median, IVW, simple mode, and weighted mode) are presented for comparison.
(PDF)

S1 Table Powers of GIVs in cohort 1. (XLSX)

S2 Table Powers of GIVs in cohort 2. (XLSX)

S3 Table Powers of GIVs in cohort 3. (XLSX)

S4 Table Powers of GIVs in cohort 4. (XLSX)

S5 Table The reversed MR results of LTL on risk of PCa. (XLSX)

S1 File The data and R code used for MR analysis. (ZIP)

## Abbreviations

PCa: prostate cancer
MR: Mendelian randomization
SNPs: single-nucleotide polymorphisms
LTL: leukocyte telomere length
GWAS: genome-wide association study
IVW: inverse-variance-weighted
OR: odds ratio
CI: confidence interval
GIV: genetic instrument variables
PRESSO: pleiotropy residual sum outlier
HR: hazard ratio
