# Supplementary figures and images for: Mendelian randomization study on the causal relationship between leukocyte telomere length and prostate cancer
Source: PLoS One. 2023 Jun 23;18(6):e0286219. doi: 10.1371/journal.pone.0286219 (PMC10289467; doi:10.1371/journal.pone.0286219)

A

Cohort 1

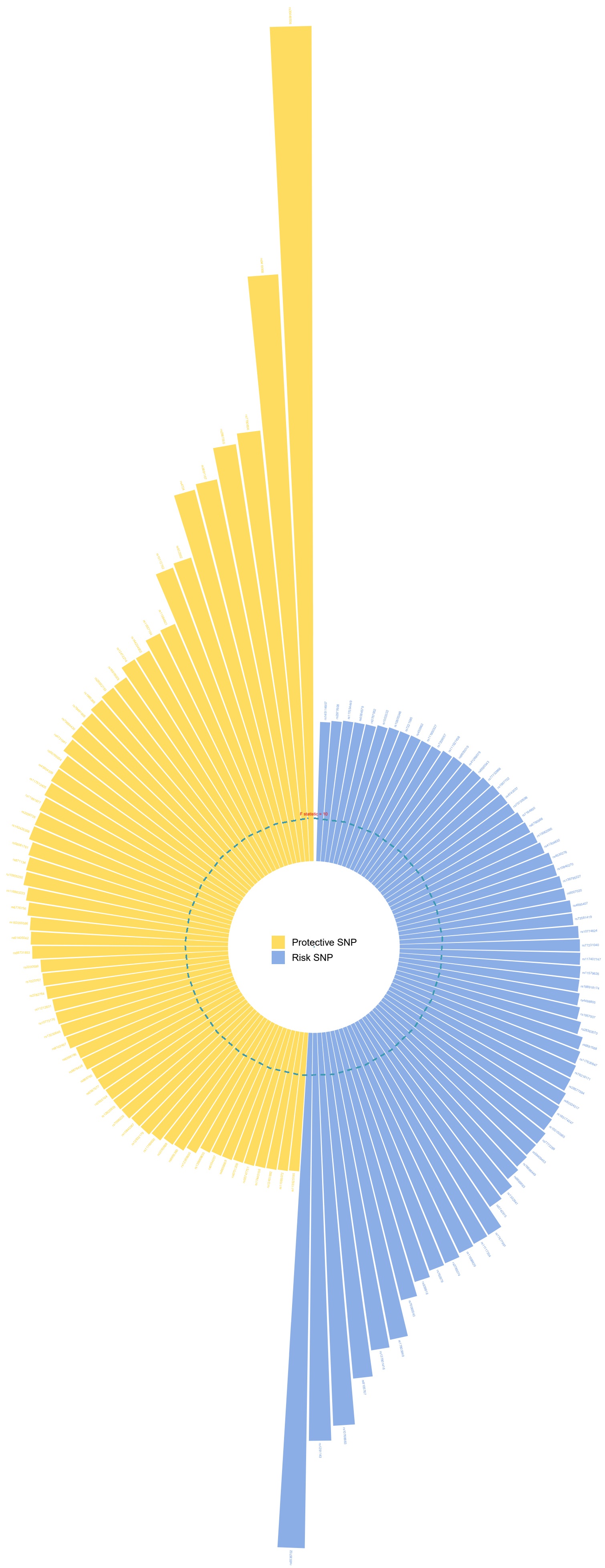

B

Cohort 2

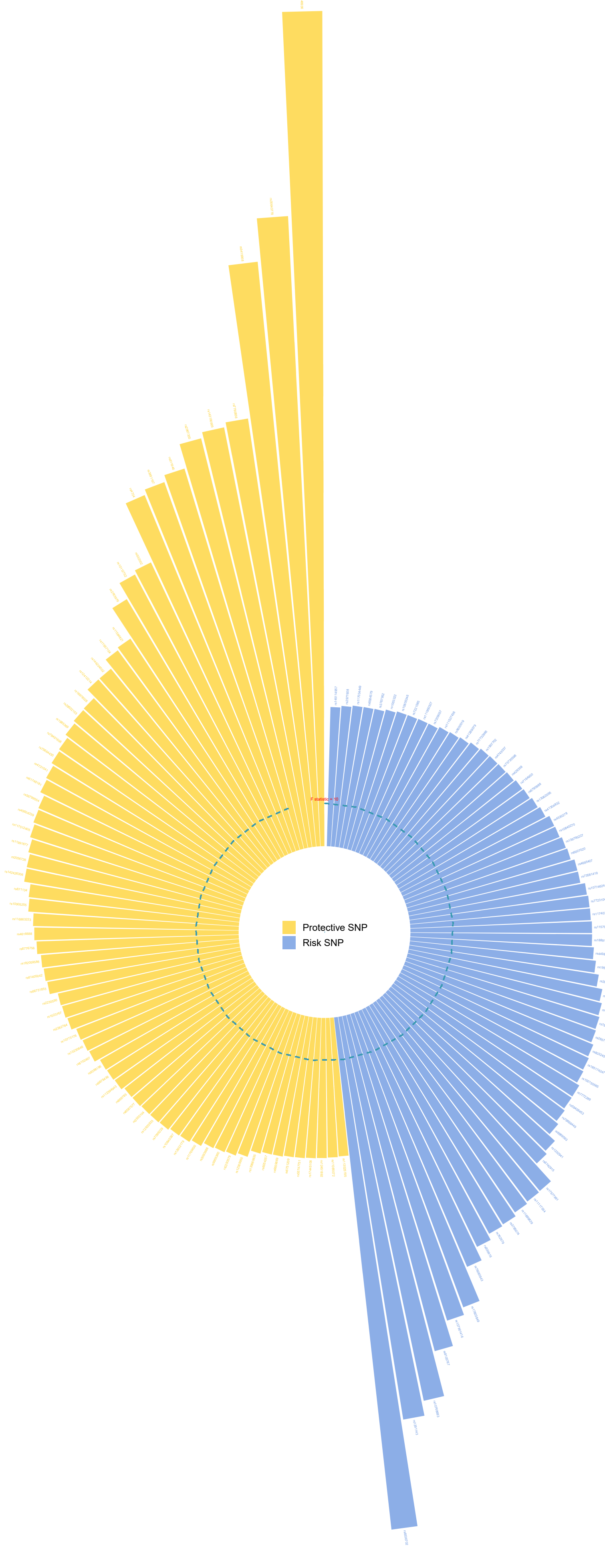

C

Cohort 3

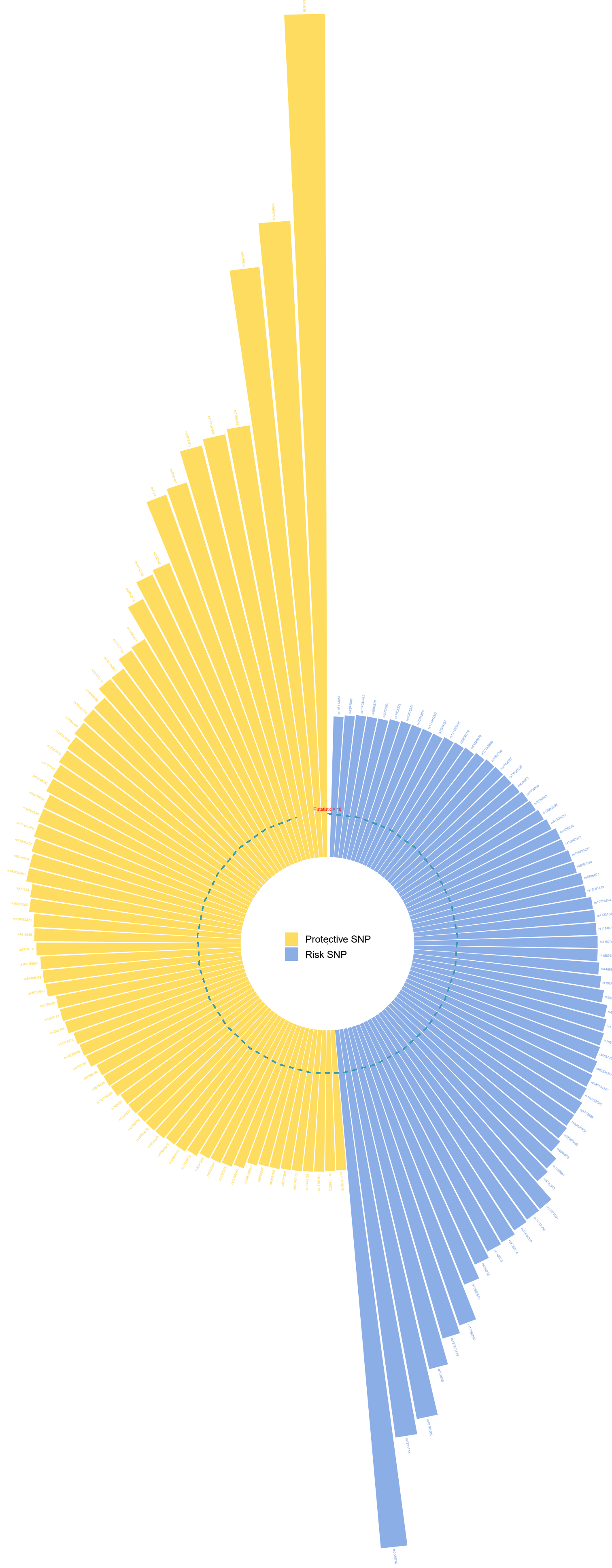

D

Cohort 4

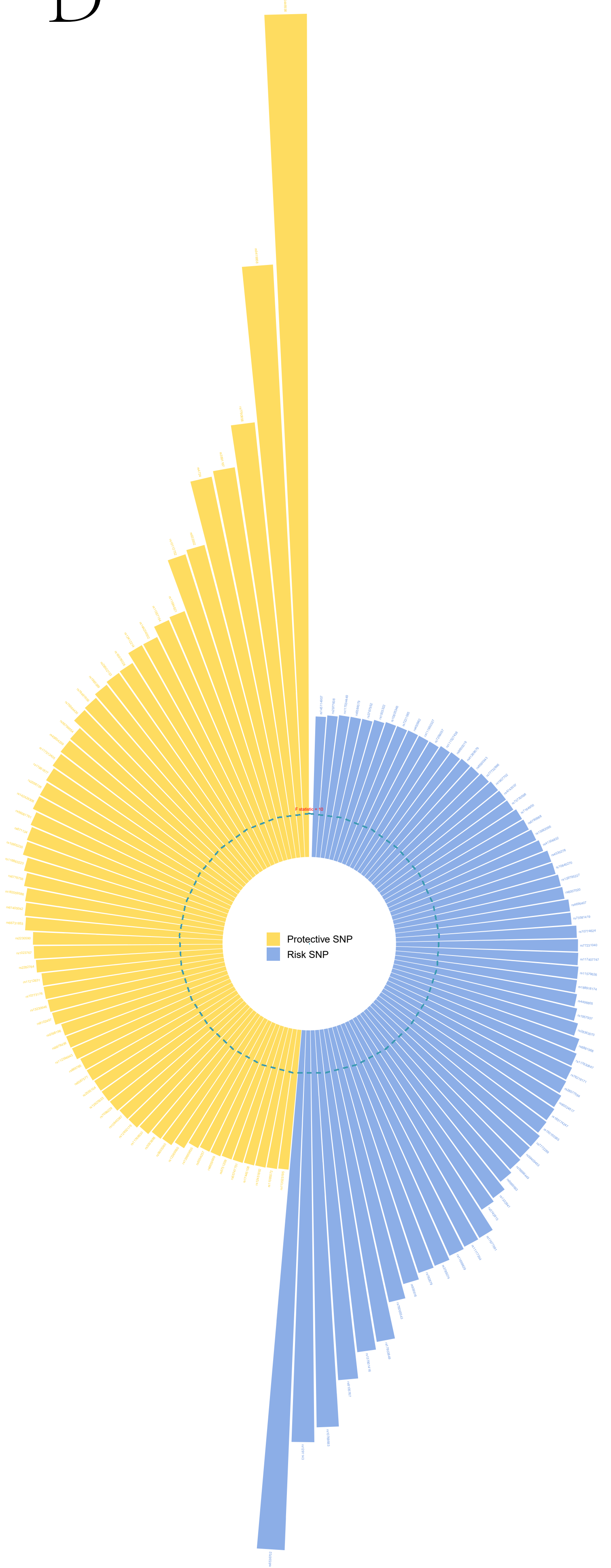

Supplement: S1 Fig — A–D, Results of F statistics of the four cohorts. (PDF) [file pone.0286219.s001.pdf]

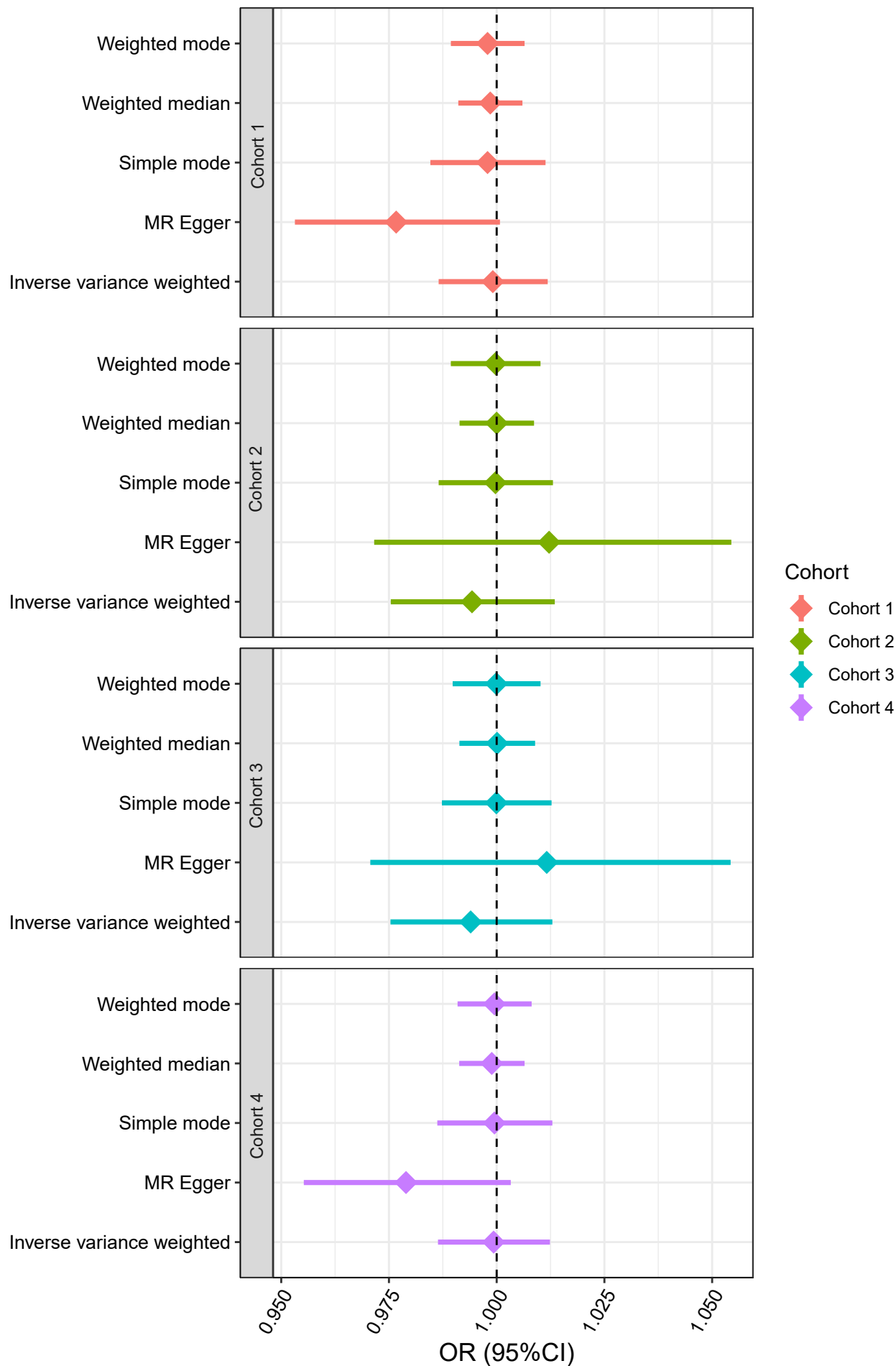

Supplement: S3 Fig — Presented OR and CI correspond to the effects of PCa on LTL (four cohorts). Results of MR using various analysis methods (MR-Egger, weighted median, IVW, simple mode, and weighted mode) are presented for comparison. (PDF) [file pone.0286219.s003.pdf]
