# Supplementary material for: Mendelian randomization study on the causal relationship between leukocyte telomere length and prostate cancer
Source: PLoS One. 2023 Jun 23;18(6):e0286219. doi: 10.1371/journal.pone.0286219 (PMC10289467; doi:10.1371/journal.pone.0286219)
Supplement: S1 File — (ZIP) [file pone.0286219.s009.zip › Rawdata_Rcode/Raw_data_csv_Files/Catalogues of Files.docx]

**File 1** Raw data for analyzing the effect of LTL on PCa risk (cohort 1).

**File 2** Raw data for analyzing the effect of LTL on PCa risk (cohort 2).

**File 3** Raw data for analyzing the effect of LTL on PCa risk (cohort 3).

**File 4** Raw data for analyzing the effect of LTL on PCa risk (cohort 4).

**File 5** Raw data for analyzing the effect of PCa risk (cohort 1) on LTL.

**File 6** Raw data for analyzing the effect of PCa risk (cohort 2) on LTL.

**File 7** Raw data for analyzing the effect of PCa risk (cohort 3) on LTL.

**File 8** Raw data for analyzing the effect of PCa risk (cohort 4) on LTL.
