# Supplementary material for: Mendelian randomization study on the causal relationship between leukocyte telomere length and prostate cancer
Source: PLoS One. 2023 Jun 23;18(6):e0286219. doi: 10.1371/journal.pone.0286219 (PMC10289467; doi:10.1371/journal.pone.0286219)
Supplement: S2 Fig — Leave-one-out analysis for IVW MR of LTL on PCa in summary-level analyses. SNP = single-nucleotide polymorphism; LTL = leukocyte telomere length; PCa = prostate cancer; MR = Mendelian randomization. (PDF) [file pone.0286219.s002.pdf]

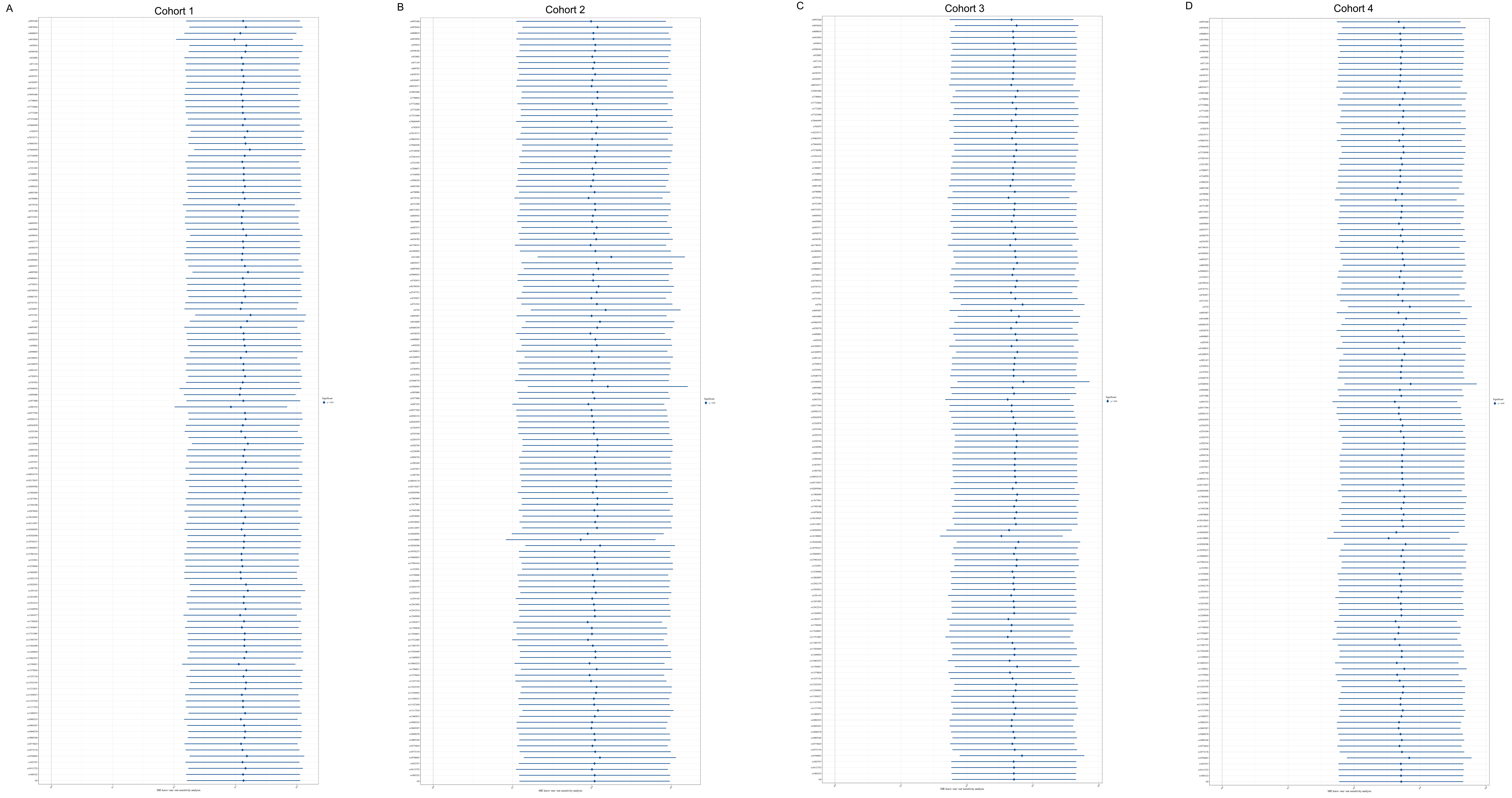
A
Cohort 1
B
Cohort 2
C
Cohort 3
D
Cohort 4
